# Deoxyribonuclease 1 Q222R single nucleotide polymorphism and long-term mortality after acute myocardial infarction

**DOI:** 10.1007/s00395-021-00864-w

**Published:** 2021-04-23

**Authors:** Thomas M. Hofbauer, Andreas Mangold, Anna S. Ondracek, Adelheid Panzenböck, Thomas Scherz, Julian Müller, Klaus Distelmaier, Veronika Seidl, Stefan Kastl, Martina Müller-Nurasyid, Annette Peters, Konstantin Strauch, Robert Winker, Evelyne Wohlschläger-Krenn, Sonja Nistler, Irene M. Lang

**Affiliations:** 1 Department of Cardiology, Division of Cardiology, Medical University of Vienna, Währinger Gürtel 18-20, 1090 Vienna, Austria; 2 Institute of Genetic Epidemiology, Helmholtz Zentrum München - German Research Center for Environmental Health, Neuherberg, Germany; 3 IBE (Institute for Medical Information Processing, Biometry, and Epidemiology), Faculty of Medicine, LMU Munich, Munich, Germany; 4 Department of Internal Medicine I (Cardiology), Hospital of the Ludwig-Maximilians-University (LMU) Munich, Munich, Germany; 5 Institute of Epidemiology, Helmholtz Zentrum München - German Research Center for Environmental Health, Neuherberg, Germany; 6 Chair of Genetic Epidemiology, Faculty of Medicine, IBE, LMU Munich, Munich, Germany; 7 Institute of Medical Biostatistics, Epidemiology and Informatics (IMBEI), University Medical Center, Johannes Gutenberg University, Mainz, Germany; 8 Health and Prevention Center, Sanatorium Hera, Löblichgasse 14, 1090 Vienna, Austria

**Keywords:** ST-segment elevation myocardial infarction, Neutrophil extracellular traps, Deoxyribonuclease, Single nucleotide polymorphism, Mortality

## Abstract

Upon activation, neutrophils release neutrophil extracellular traps (NETs), which contribute to circulating DNA burden and thrombosis, including ST-segment elevation myocardial infarction (STEMI). Deoxyribonuclease (DNase) 1 degrades circulating DNA and NETs. Lower DNase activity correlates with NET burden and infarct size. The DNase 1 Q222R single nucleotide polymorphism (SNP), impairing DNase 1 function, is linked with myocardial infarction. We assessed whether the Q222R SNP is connected to increased NET burden in STEMI and influences long-term outcomes. We enrolled 711 STEMI patients undergoing primary percutaneous coronary intervention (pPCI), and 1422 controls. Genotyping was performed for DNase 1 Q222R SNP. DNase activity, double-stranded (ds)DNA and citrullinated histone H3 were determined in culprit site and peripheral plasma during pPCI. The association of the Q222R variant on cardiovascular and all-cause mortality was assessed by multivariable Cox regression adjusted for cardiovascular risk factors. Homozygous Q222R DNase 1 variant was present in 64 (9.0%) STEMI patients, at the same frequency as in controls. Patients homozygous for Q222R displayed less DNase activity and increased circulating DNA burden. In overall patients, median survival was 60 months. Homozygous Q222R variant was independently associated with cardiovascular and all-cause mortality after STEMI. dsDNA/DNase ratio independently predicted cardiovascular and all-cause mortality. These findings highlight that the Q222R DNase 1 SNP is associated with increased NET burden and decreased compensatory DNase activity, and may serve as an independent risk factor for poor outcome after STEMI.

**Supplementary Information:**

The online version contains supplementary material available at 10.1007/s00395-021-00864-w.

## Introduction

ST-segment elevation myocardial infarction (STEMI) is a substantial health burden [17]. Atherothrombosis and ischemia/reperfusion during STEMI are linked to leukocyte activation [27]. Innate immune cells, especially neutrophils, are recruited early to sites of tissue injury and inflammation [48].

Apart from degranulation and phagocytosis, neutrophils are able to release their nuclear content into the extracellular space by formation of neutrophil extracellular traps (NETs), resulting in DNA webs interspersed with histones and granular proteins [7]. NETs participate in host defense [6] and have highly pro-inflammatory [29], cytotoxic [40] and pro-thrombotic properties [14], in STEMI [30] and ischemic stroke [26]. Even in the absence of platelets and fibrin, NETs form potentially lethal vascular occlusions in vivo [22].

Neutrophils are attracted to the culprit site of STEMI patients and release high levels of NETs that have been associated with increased infarct size [30]. Recently, we have also reported that components of NETs promote activation and differentiation of fibrocytes after STEMI, initiating adverse remodeling of the affected myocardium [18, 31]. Thereby, we complemented data of previous studies, suggesting that NETs are mediators of vascular healing and fibrosis [32]. NETs significantly contribute to circulating DNA burden, which is connected to poor prognosis after myocardial infarction [28, 49].

NETs can be degraded by deoxyribonuclease (DNase) hydrolyzing the backbone of dsDNA. Deoxyribonuclease (DNase) 1 and 1L3 are the major enzymes which degrade NETs in the circulation [35]. It was shown that intact plasmatic DNase function is critical in neutrophilic mice to maintain the balance between NET formation vs. degradation, and survival [22]. Correspondingly, DNase deficiency has been associated with disease in humans, including systemic lupus erythematosus [52] and thrombotic microangiopathy [21]. DNase 1 treatment in rodent myocardial infarction models resulted in markedly reduced infarct size [42, 47]. In STEMI patients, decreased DNase activity at the culprit site was associated with higher thrombus NET burden and bigger infarct size [30].

Factors influencing chromatin degradation in circulation by DNase are enzyme release, substrate concentration, pH, inhibitory or stimulating co-factors and genetic background. Two phenotypes are described for DNase 1, which are discriminated by a single nucleotide polymorphism (SNP): Presence of adenine instead of guanine at chromosome 16:3657746 results in an amino acid substitution of arginine by glutamine at position 222 (Q222R) of the enzyme. The Q222R variant leads to impaired enzymatic activity [51] and was associated with increased prevalence of MI in a Japanese cohort [25].

We hypothesized that the Q222R SNP is connected with decreased survival after STEMI by impaired DNase activity and increased extracellular DNA burden.

## Methods

### STEMI patients and controls

Between 2006 and 2016, we recruited consecutive patients (*n* = 711) undergoing primary percutaneous intervention for STEMI, with a door-to-balloon time below 120 min. Inclusion and exclusion criteria were applied, and intervention was performed as previously described [18, 30]. Inclusion criteria were (1) chest pain at coronary angiography, (2) new ST-segment elevations of ≥ 2 mm on > 1 chest lead or new ST elevations of ≥ 1 mm on > 1 limb lead within 20 min of coronary angiography, and (3) coronary anatomy suitable for thrombectomy. Patients under immunosuppression or treatment with glycoprotein IIb/IIIa-blockers were excluded. All patients were heparinized at an activated coagulation time of > 300 s (4000–10,000 IE) and received 250 mg of acetylsalicylic acid.

Thrombectomy was performed when the following general criteria were fulfilled: vessel diameter ≥ 3 mm; large intraluminal contrast medium filling defect; thrombus located within 50 mm of respective coronary ostium; absence of severe tortuosity; non-difficult vascular access. Via a thrombectomy catheter, 10 to 20 ml of culprit site blood was collected into tubes containing EDTA. Peripheral sheath blood served as control. To account for dilution due to flushing of catheters, concentrations were normalized to the hematocrit difference between culprit and peripheral site sample. Blood was immediately centrifuged for 10 min, 2000 g at 21 °C, and plasma and buffy coats were stored at − 80 °C for subsequent analyses.

We included age- and sex-matched clients without a history of MI (*n* = 1422) recruited at the Health and Prevention Center, Sanatorium Hera, Vienna, Austria and the Helmholtz Zentrum München, Munich, Germany [19] as controls.

### Enzymatic infarct size

Enzymatic infarct size was computed employing the trapezoidal formula for the area under the curve of creatine-phosphokinase isoform MB (CK-MB AUC) as previously described [10], and expressed as arbitrary units.

### ST-segment resolution

ST-segment resolution (STR) was calculated as previously described [8]. ST-segment elevation was measured at the J point in surface lead electrocardiograms (in case of anterior infarction: I, aVL, V1–V6; in case of inferior infarction: II, III, aVF, V5, V6. Measurements were performed in the index electrocardiogram and 30 min after pPCI. ST-segment elevation was calculated as a ratio and is given in percent (hence, 100% indicates complete resolution of ST-segment elevation).

### Transthoracic echocardiography after STEMI

Complete echocardiographic exams were performed from STEMI patients in the course of routine care by experienced observers on a GE Vivid E9 or a GE Vivid E95. Echocardiography was performed at a median follow-up of 3 [IQR 2, 4] days after STEMI. The following parameters were evaluated in the present study, if an exam within 30 days after pPCI in sufficient quality was available: left ventricular end-systolic volume (ESV), left ventricular end-diastolic volume (EDV), left ventricular ejection fraction (LVEF) and left ventricular global longitudinal strain (GLS). Overall, data from 455 patients was obtained. Biplane left ventricular ejection fraction was calculated employing the Simpson method in 371 patients as a measure of cardiac function after STEMI [9]. Global longitudinal strain was measured in 208 patients. Speckle tracking analysis was performed in three apical views (long-axis view, four chamber view and two chamber view) using specific software (EchoPacs, GE Healthcare). Global longitudinal strain was computed as the mean of the global peak systolic strain in each view as an additional measure of cardiac function after STEMI [23].

### Isolation of genomic DNA

Genomic DNA was isolated from buffy coats using a ReliaPrep™ Blood gDNA Miniprep System (Promega, A5082) according to manufacturer’s instructions. 200 µl of sample were mixed with cell lysis buffer and proteinase K and incubated at 56 °C for 10 min. Lysates were transferred onto binding columns and centrifuged for 1 min at 14,000×*g*. Columns were washed three times with provided washing solution. Then, membrane-bound DNA was eluted in 50 µl nuclease-free water. Concentration and quality of DNA were assessed using a NanoDrop 1000 spectrophotometer (ND1000, PeqLab).

### Genotyping of SNPs

For determination of the Q222R DNase 1 SNP (rs1053874 [46]), a TaqMan™ allelic discrimination assay (ThermoFisher, 4351379) and GoTaq® Probe qPCR master mix (Promega, A6102) were used according to manufacturer’s instructions and performed in duplicates. Fluorescence was measured, and the genotype was calculated, using a 7500 Real Time PCR System (Applied Biosystems, Software Version 2.3).

### Measurement of DNase activity

Total DNase activity was measured using single radial enzyme diffusion technique as previously described [22], with modifications. Salmon testes DNA (Sigma-Aldrich, D1626-1G) was dissolved at a concentration of 100 µg/ml in assay buffer containing divalent cations and a DNA-binding fluorescent dye (35 mM Tris–HCl, pH 7.8, 20 mM MgCl_2_, 2 mM CaCl_2_, 2.5 × SYBR Safe [Invitrogen, S33102]) as substrate for DNases. After heating the solution to 50 °C for 10 min, an equal volume of 2% ultra-pure agarose (Invitrogen, 16500–500) was added. The mixture was poured into a plastic tray to allow solidification. Then, 2 µl sample or standard were loaded into wells with a diameter of 1 mm and incubated at 37 °C for 20 h. Remaining fluorescence of gels was recorded using Biorad ChemiDoc XRS + fluorescence scanner. DNase activity was calculated according to a standard curve (Dornase alfa, Roche). DNase activity was measured by an investigator blinded to genotypes.

### Measurement of soluble NET markers

Double-stranded DNA (dsDNA) was detected using Sytox Green (ThermoFisher, P7020) as previously described [20]. Sytox Green, a fluorescent dye staining cell-free dsDNA, was added to plasma samples diluted 1:20 or standard (lambda DNA, ThermoFisher, P7589) for 5 min, after which fluorescence was measured using a Synergy H1 Hybrid microplate reader (BioTek, excitation 480 nm, emission 520 nm). Fluorescence intensities were normalized to the standard curve.

Citrullinated histone H3 (citH3) was measured as previously described [45], with minor modifications [18]. Washing steps were performed with phosphate-buffered saline (PBS) containing 0.05% Tween-20. Anti-histone biotin provided in the Cell Death Detection ELISA PLUS Kit (Roche, 11 774 425 001) was incubated in streptavidin-coated 96-well plates for 2 h. Wells were incubated with 50 µl of undiluted plasma samples or citH3 standard (Cayman, 17926) for 1.5 h. Plates were then incubated with anti-citrullinated histone H3 (1:2000 in PBS + 1% bovine serum albumin [BSA]) for 1 h, after which horseradish peroxidase conjugate antibody (1:5000 in PBS + 1% BSA, BioRad, 170–6515) was added for 1 h. TMB Substrate solution (ThermoFisher, N301) was used to start the enzymatic reaction. After 20 min, the reaction was stopped by addition of 2 M H_2_SO_4_. Optical density was measured on a Promega GloMax Discover microplate reader (450 nm, reference 620 nm) and normalized to standard curve.

### Outcome assessment

Mortality data was obtained from the Austrian Registry of Death (Statistics Austria, Vienna, Austria). This registry is prospectively updated on an annual basis, including every Austrian resident. Causes of death were classified into cardiovascular and all-cause death according to the International Statistical Classification of Disease and Related Health Problems, 10th revision (ICD-10).

### Statistical analysis

Given the cross-sectional nature of our study, and because no preliminary data on the influence of a DNase 1 SNP on mortality was available at the start of the study, no sample size calculation was performed. Patient recruitment was performed over a period of 10 years. With a sample size of 711 patients, a hazard ratio (HR) of 2, overall mortality of 18.7% and a frequency of a homozygous DNase 1 variant of 9.3%, the power was 0.65. We defined all-cause and cardiovascular mortality as primary endpoints. Chi square statistics were used to analyze for a difference in allele frequency between groups. Normality of data was assessed using the Kolmogorov–Smirnov test and histograms. Data are given as median and interquartile range (IQR). Comparisons between two groups were performed using Wilcoxon signed-rank test for paired data, or Mann Whitney test for unpaired data. Three groups were compared by one-way analysis of variance with Dunn’s multiple post-hoc comparison. By multivariable Cox regression, we assessed the influence of 1) Q222R DNase 1 SNP; and 2) the ratio between dsDNA and DNase activity divided by the standard deviation; on cardiovascular and all-cause mortality. Data are given as HR and 95% confidence interval (CI). We adjusted for the following established cardiovascular risk factors: age, male sex, body mass index (BMI), hyperlipidemia, arterial hypertension, diabetes mellitus, ever smoker and renal function as measured by serum creatinine concentration on admission. Alpha < 0.05 was considered statistically significant. All statistical analyses were performed using SPSS 25.0 (IBM). Figures were generated using GraphPad Prism 8. Whiskers of box plots were defined according to Tukey’s method. Outliers are presented as dots.

## Results

### Patient characteristics

We studied 711 patients presenting with STEMI and angiographic TIMI flow of 0–1 in the culprit vessel. Median survival after STEMI was 60.0 [IQR 30.3, 91.5] months. While a total of 133 (18.7%) patients died, 78 (11.0%) deaths were assigned to cardiovascular causes. Detailed patient characteristics are shown in Table 1. Characteristics of controls are shown in Supplementary Table 1.

**Table 1:** Baseline characteristics of STEMI patients

	Patient characteristics (*n* = 711)
Age in years, median [IQR]	58 [49, 68]
Male sex, No. (%)	551 (77.5)
BMI, median [IQR]	27.4 [24.8, 30.1]
BMI > 25 kg/m^2^, No. (%)	525 (73.8)
BMI > 30 kg/m^2^, No. (%)	182 (25.6)
Diabetes, No. (%)	142 (20.0)
History of hypertension, No. (%)	490 (68.9)
Dyslipidemia, No. (%)	481 (67.7)
Ever smoker, No. (%)	490 (68.9)
Family history of CAD, No. (%)	246 (34.6)
Previous MI, No. (%)	137 (19.3)
Culprit lesion, No. (%)	
LAD	319 (44.9)
CX	102 (14.3)
RCA	262 (36.9)
Multiple	10 (1.4)
Bypass	18 (2.5)
CAD, No. (%)	
1VD	403 (56.7)
2VD	180 (25.3)
3VD	128 (18.0)
In-stent restenosis, No. (%)	63 (8.9)
CRP, mg/dL (< 0.5), median [IQR]	0.38 [0.18, 0.77]
Creatinine on admission, mg/dL (0.7–1.3), median [IQR]	1.21 [1.05, 1.39]
Cholesterol, mg/dL (< 200), median [IQR]	199.61 [170.66, 232.82]
LDL, mg/dL (< 130), median [IQR]	113.13 [88.42, 140.15]
HDL, mg/dL (> 55), median [IQR]	40.93 [35.14, 49.03]
Triglycerides, mg/dL (< 150), median [IQR]	122.12 [81.42, 192.04]
CK-MB AUC, median [IQR]	213.3 [95.5, 404.5]
STR, %, median [IQR]	70.0 [38.4, 87.8]
Transthoracic echocardiography after STEMI	365 (51.3)
Days after STEMI, median [IQR]	3 [2, 4]
LVEF, %, median [IQR]	49 [44, 56]
ESV, ml, median [IQR]	49 [37, 66]
EDV, ml, median [IQR]	98 [81, 120]
GLS, %, median [IQR]	− 14.1 [− 16.8, − 11.0]
Background medication prior to admission	
ACE inhibitors, No. (%)	115 (16.2)
ARB, No. (%)	72 (10.1)
Beta-blockers, No. (%)	138 (19.4)
Statins, No. (%)	140 (19.7)
ASA, No. (%)	166 (23.3)

Data are given as median [IQR] or percent of patients. Values in parentheses indicate reference values. Conversion factor for SI units for CRP 10; creatinine 76.25; cholesterol, LDL and HDL 0.0259; triglycerides 0.0113

*ACE-I* angiotensin converting enzyme inhibitor, *ARB* angiotensin receptor blocker, *ASA* acetylsalicylic acid, *AUC* area under the curve, BMI body mass index, *CAD* coronary artery disease, *CK-MB* creatine-phosphokinase isoform MB, *CRP* C-reactive protein, *CX* circumflex artery, *EDV* end-diastolic volume, *ESV* end-systolic volume, *GLS* global longitudinal strain, *HDL* high-density lipoprotein, *IQR* interquartile range, *LAD* left anterior descending artery, *LDL* low-density lipoprotein, *LVEF* left ventricular ejection fraction, *MI* myocardial infarction, *MRA* mineralocorticoid receptor antagonists, *RCA* right coronary artery, *STR* ST-segment resolution, *SV* stroke volume, *VD* vessel disease

### Frequency of DNase 1 Q222R SNP

**Fig. 1:**
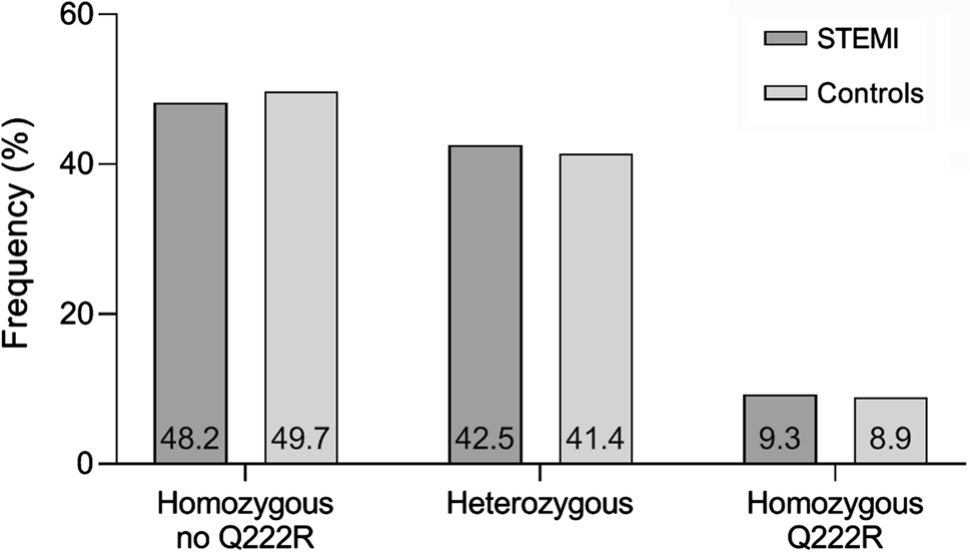
Overall allele frequency of Q222R DNase 1 SNP in STEMI patients and non-MI controls. Frequency of the Q222R DNase 1 SNP in STEMI patients (*n* = 711) and an age- and sex-matched cohort of patients without history of MI (*n* = 1422). Numbers in bars indicate the percentage of patients per group. Chi square for comparison between patient cohorts was χ^2^ = 0.336, *p* = 0.845. *DNase* deoxyribonuclease, *SNP* single nucleotide polymorphism, *STEMI* ST-segment elevation myocardial infarction

Using allelic discrimination, we assessed the frequency of the Q222R DNase 1 SNP in STEMI patients (*n* = 711, Fig. 1). The homozygous variant was present in 66 (9.3%) patients; 302 (42.5%) were heterozygous, and 343 (48.2%) did not carry the variant of interest.

The relative frequency of the Q222R DNase 1 SNP in STEMI was compared with a Caucasian age- and sex-matched control cohort (*n* = 1422, Fig. 1). In this cohort, 126 (8.9%) patients presented with the homozygous deficient variant, while 589 (41.4%) and 702 (49.7%) were heterozygous and homozygous for the other allele, respectively. No difference in SNP frequency was found between the STEMI and control cohorts (Chi square *χ*^2^ = 0.336, *p* = 0.845). Furthermore, no differences in age between STEMI patients and non-MI controls were present, irrespective of the genotype (58 [49, 68] vs. 58 [49, 68] years, *p* = 0.152).

### NET markers are increased at the culprit site, while DNase activity is low in overall patients

**Fig. 2:**
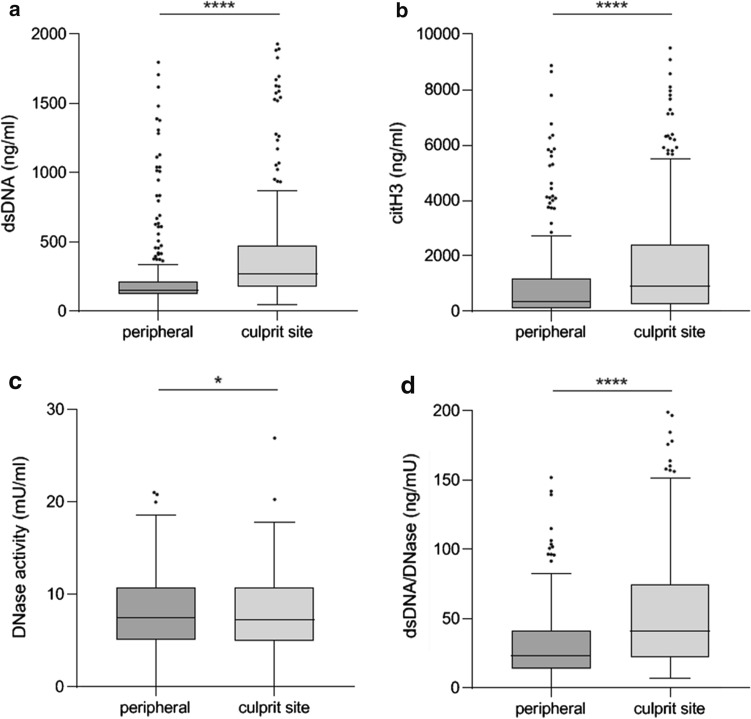
NET burden and DNase activity in STEMI patients. a, dsDNA (*n* = 315, culprit site 270.6 [178.4, 478.2] vs. peripheral 152.2 [123.9, 218.2] ng/ml, *p* < 0.0001). 4 peripheral and 10 culprit site values are out of y-axis range. b, citH3 (*n* = 342, culprit site 897.3 [259.3, 2423.0] vs. peripheral 453.7 [110.3, 1182.0] ng/ml, *p* < 0.0001). 12 peripheral and 18 culprit site values are out of y-axis range. c, NE (*n* = 226, culprit site 119.2 [61.3, 237.1] vs. peripheral 38.7 [16.7, 78.4] ng/ml, *p* < 0.0001). 2 culprit site value are out of y-axis range. d, MPO (*n* = 226, culprit site 265.6 [223.2, 311.0] vs. peripheral 233.7 [207.8, 251.2] ng/ml, *p* > 0.0001). 1 culprit site value is out of y-axis range. e, DNase activity (*n* = 225, culprit site 7.29 [5.00, 10.80] vs. peripheral 7.46 [5.12, 10.80] mU/ml, *p* = 0.021). f, ratio between dsDNA and DNase activity (*n* = 227, culprit site 41.27 [22.56, 74.59] vs. peripheral 23.30 [13.78, 41.43] ng/mU, *p*< 0.0001). 6 peripheral and 4 culprit site values are out of y-axis range. *p < 0.05, ****p < 0.0001. citH3 citrullinated histone H3, DNase deoxyribonuclease, dsDNA double-stranded DNA, MPO myeloperoxidase, NE neutrophil elastase, STEMI ST-segment elevation myocardial infarction. Two-sided Wilcoxon signed-rank test, alpha-level 0.05

We compared dsDNA, citH3 and DNase activity between the culprit and peripheral site of STEMI patients. dsDNA, a non-specific marker of NETosis and cell death [38], was higher at the culprit site than at the peripheral site (Fig. 2a), as shown before [18, 30, 43]. citH3, a specific marker of NETs [26, 50], was highly increased at the culprit site (Fig. 2b). Also, both NE (Fig. 2c) and MPO (Fig. 2d) were increased at the culprit site. We found positive correlations between dsDNA, citH3, NE and MPO, both at the culprit (Supplementary Table 2) and peripheral site (Supplementary Table 3). DNase activity was significantly higher in STEMI patients than in controls (Supplementary Fig. 1a). DNase activity was lower at the culprit site (Fig. 2e), while dsDNA to DNase activity ratio was higher at the culprit site than at the peripheral site (Fig. 2f).

**Table 2:** Effect of the homozygous Q222R DNase 1 SNP on cardiovascular and all-cause mortality of STEMI patients at long-term follow-up

Factor	Cardiovascular mortality			All-cause mortality		
	Adjusted HR	95% CI	*p*-value	Adjusted HR	95% CI	*p*-value
Age	1.09	1.06–1.12	< 0.0001	1.07	1.05–1.09	< 0.0001
Male sex	0.99	0.56–1.77	0.975	1.29	0.84–1.99	0.246
BMI	1.02	0.97–1.08	0.440	0.99	0.94–1.03	0.531
Hyperlipidemia	0.73	0.43–1.25	0.248	0.68	0.46–1.02	0.061
Arterial hypertension	0.72	0.34–1.37	0.322	0.55	0.35–0.87	0.010
Diabetes mellitus	2.57	1.50–4.41	0.001	2.28	1.50–3.47	< 0.0001
Ever smoker	0.66	0.38–1.13	0.127	0.85	0.57–1.29	0.450
Creatinine on admission	2.63	1.83–3.78	< 0.0001	2.84	2.15–3.76	< 0.0001
DNase 1 SNP	2.02	1.01–4.01	0.046	2.01	1.91–3.39	0.009

Multivariable Cox regression was used to assess the influence of a homozygous SNP in the Q222R DNase 1 gene on cardiovascular and all-cause mortality, after a median follow-up of 60.0 [IQR 30.3, 91.5] months

*BMI* body mass index, *CI* confidence interval, *DNase* deoxyribonuclease, *HR* hazard ratio, *IQR* interquartile range, *SNP* single nucleotide polymorphism, *STEMI* ST-segment elevation myocardial infarction

**Table 3:** Effect of the dsDNA to DNase activity ratio measured at the peripheral site on cardiovascular and all-cause mortality of STEMI patients at long-term follow-up

Factor	Cardiovascular mortality			All-cause mortality		
	Adjusted HR	95% CI	*p*-value	Adjusted HR	95%	*p*-value
Age	1.10	1.03–1.18	0.003	1.01	1.05–1.14	< 0.0001
Male sex	0.47	0.92–2.37	0.357	1.26	0.49–3.22	0.635
BMI	0.89	0.76–1.05	0.179	0.95	0.86–1.06	0.374
Hyperlipidemia	0.43	0.12–1.58	0.204	0.79	0.33–1.89	0.600
Arterial hypertension	0.38	0.10–1.47	0.162	0.26	0.11–0.63	0.003
Diabetes mellitus	4.84	1.30–18.07	0.019	2.19	0.80–6.04	0.128
Ever smoker	1.13	0.28–4.56	0.863	1.72	0.67–4.47	0.262
Creatinine on admission	4.02	1.63–9.89	0.003	2.94	1.43–6.05	0.003
dsDNA/DNase activity ratio	1.28	1.05–1.57	0.016	1.25	1.05–1.48	0.012

Multivariable Cox regression was used to assess the influence of a homozygous SNP in the Q222R DNase 1 gene on cardiovascular and all-cause mortality, after a median follow-up of 60.0 [IQR 30.3, 91.5] months

*BMI* body mass index, *CI* confidence interval, *DNase* deoxyribonuclease, *dsDNA* double-stranded DNA, *HR* hazard ratio, *IQR* interquartile range, *SNP* single nucleotide polymorphism, *STEMI* ST-segment elevation myocardial infarction.

### Homozygous DNase 1 Q222R SNP leads to decreased enzymatic activity

**Fig. 3:**
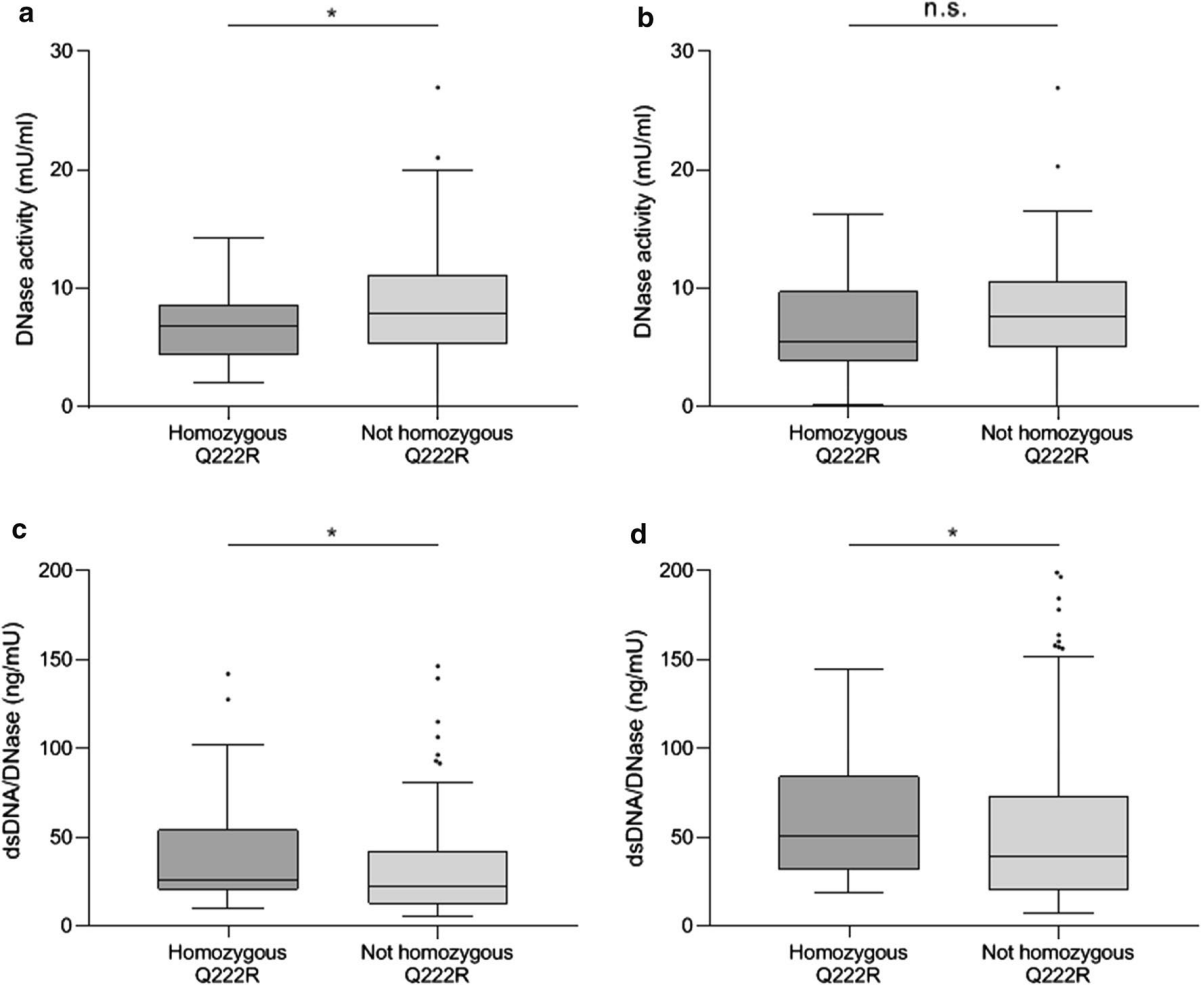
DNase activity and dsDNA levels in STEMI patients with homozygous DNase 1 Q222R. DNase activity at a, the peripheral site (homozygous Q222R *n* = 42, 6.76 [4.38, 8.67] vs. not homozygous Q222R *n* = 176, 7.91 [5.28, 11.18] mU/ml, *p* = 0.047) and b, the culprit site (homozygous Q222R *n* = 34, 5.49 [3.86, 9.73] vs. not homozygous Q222R *n* = 153, 7.57 [5.00, 10.64] mU/ml, *p* = 0.100) of STEMI patients was measured in patients with or without a homozygous variant of the Q222R DNase 1 SNP. dsDNA at c, the peripheral site (homozygous Q222R *n* = 40, 26.31 [20.29, 54.26] vs. not homozygous Q222R *n* = 174, 22.48 [12.70, 42.40] mU/ml, *p* = 0.030) and d, the culprit site (homozygous Q222R *n* = 33, 50.68 [31.69, 84.77] vs. not homozygous Q222R *n* = 153, 39.43 [20.46, 73.54] mU/ml, *p* = 0.027) was measured and divided by DNase activity. In c, 1 value in the homozygous and 3 values in the other group are out of y-axis range. In d, 2 and 5 values are out of y-axis range. DNase deoxyribonuclease, dsDNA double-stranded DNA, SNP single nucleotide polymorphism, STEMI ST-segment elevation myocardial infarction. *p < 0.05, *n.s.* not significant. Two-sided Mann Whitney test, alpha-level 0.05

To validate previously published findings [51], we assessed whether the presence of the Q222R DNase 1 SNP was associated with impaired DNase activity in healthy controls and STEMI patients. Homozygous SNP carriers exhibited lower enzymatic activity compared to respective heterozygous and non-carriers, regardless of the cohort (Fig. 3a, Supplementary Fig. 1b). At the culprit site, DNase enzymatic activity of STEMI patients was not different between genotypes (Fig. 3b). When we computed the ratio of dsDNA to DNase activity to estimate the degree of uncompensated NET formation, we found it increased in homozygous patients both at the peripheral (Fig. 3c) and the culprit site (Fig. 3d).

We next analyzed whether the presence of the homozygous Q222R DNase 1 SNP was associated with enzymatic infarct size and ST-segment resolution. We found that they were not different among genotypes (Supplementary Fig. 2a, b). Furthermore, we performed echocardiographic analyses 3 [IQR 2, 4] days after STEMI, finding no differences in LVEF, ESV, EDV or GLS between genotypes (Supplementary Fig. 2c–f, Supplementary Tables 4 and 5). We observed a positive correlation between LVEF and DNase activity at the culprit site (Supplementary Fig. 3).

### DNase Q222R 1 and dsDNA/DNase activity ratio predict mortality after STEMI

**Fig. 4:**
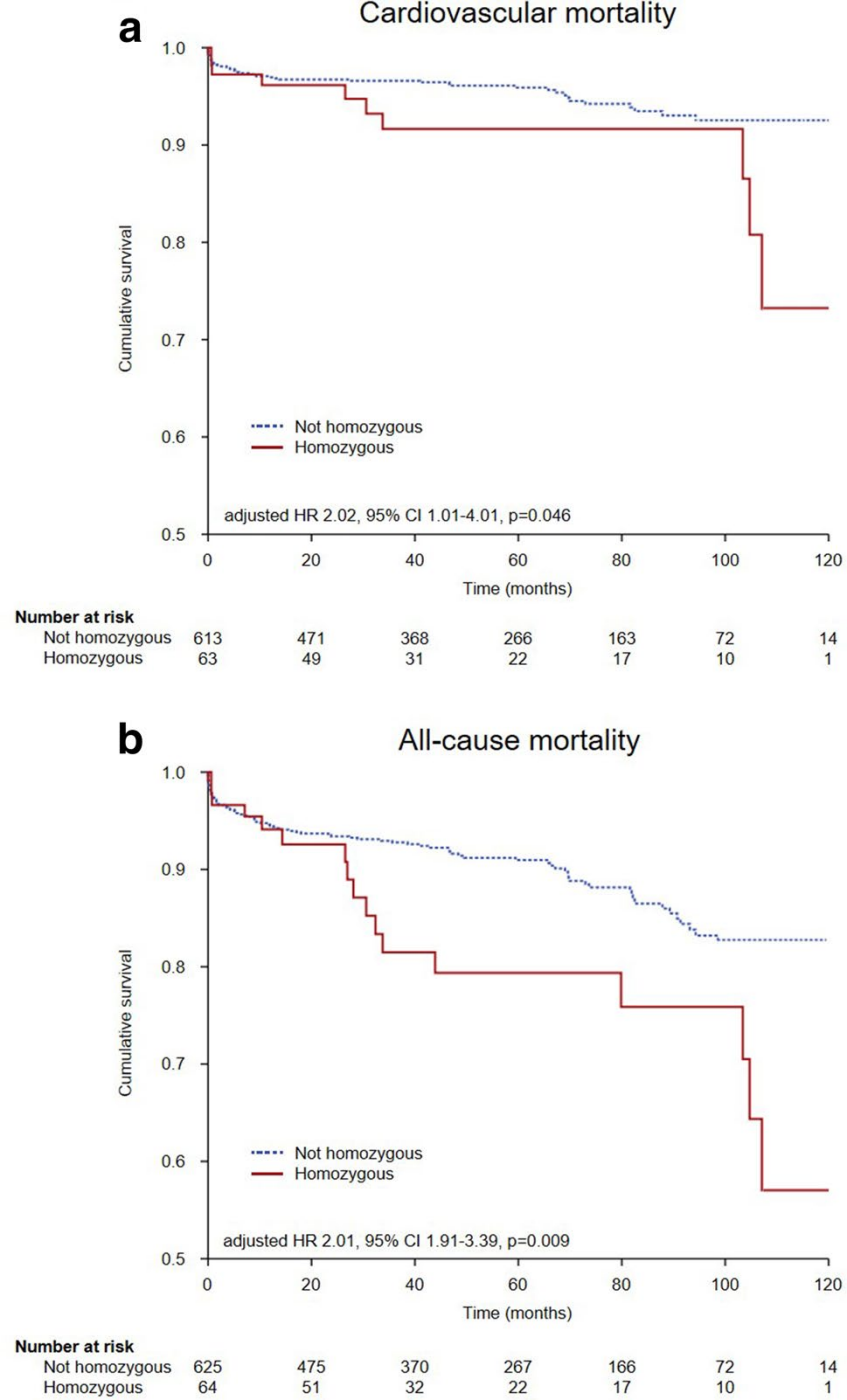
Cardiovascular and all-cause mortality of patients after STEMI with homozygous DNase 1 Q222R. Kaplan–Meier curves depicting the influence of the homozygous Q222R DNase 1 SNP on a, cardiovascular and b, all-cause mortality after STEMI. Censored patients are not shown. DNase deoxyribonuclease, SNP single nucleotide polymorphism, STEMI ST-segment elevation myocardial infarction. Multivariable Cox regression, alpha-level 0.05

To assess the influence of DNase 1 Q222R SNP on mortality, we performed multivariable Cox regression analysis, adjusting for cardiovascular risk factors. We identified the homozygous Q222R variant as independent predictor of both cardiovascular (Fig. 4a, Table 2) and all-cause mortality (Fig. 4b, Table 2). Kaplan–Meier curves stratified by all three genotypes are shown in Supplementary Fig. 4. Levels of dsDNA, citH3, NE, MPO, and DNase activity alone were not associated with outcome (data not shown). dsDNA to DNase activity ratio at the peripheral site as well as at the culprit site was independently predictive for both cardiovascular and all-cause mortality (Table 3, Supplementary Table 1).

## Discussion

In the present work we confirm increased NET burden at the coronary culprit site in a large cohort of STEMI patients, consistent with previous literature [18, 30, 43]. We show that DNase activity is a determinant of prognosis after STEMI. While the DNase 1 Q222R SNP [25] is not more common in patients than controls, it confers lower DNase 1 activity, and is an independent predictor of poor outcome after STEMI.

Previously, we have shown a high NET burden at the culprit site in STEMI [30]. Both major components of chromatin, i.e., DNA and histones mediate adverse effects. dsDNA mediates pro-thrombotic effects by activating platelets [14] and endothelial cells [15], inducing the production of pro-inflammatory cytokines. This includes interleukin-6 and monocyte chemoattractant protein-1 [34], which have been associated with poor cardiac function and outcome after STEMI [11, 16]. Histones are cytotoxic [40], and increase thrombin generation [1]. Supporting these experimental observations, NETs were shown to be an integral part of venous [5] and coronary thrombi [30, 43], serving as scaffolds for thrombosis. Increased NET burden in coronary thrombi is correlated with cardiac magnetic resonance-measured infarct size, and with poor ST-segment resolution [30]. After STEMI, culprit site dsDNA levels are predictive of major adverse cardiovascular events (MACE) at 25 month follow-up [28, 49]. NETs also promote chronic atherosclerosis as shown in several murine models [12]. In humans, NETs were detected both in the lumen of atherosclerotic vessels [33] and within plaques [39], exerting pro-thrombotic and plaque-destabilizing effects, and appear to be key factors for superficial erosion [13]. In stable coronary artery disease, circulating dsDNA levels were independently associated with disease severity assessed by coronary computed tomography angiography, and with MACE [4].

Adequate vascular DNase activity is vital to efficiently degrade extracellular chromatin, to prevent tissue damage, autoimmune effects and to maintain homeostasis [22]. Major DNA-degrading enzymes in the circulation are DNase 1 and 1L3, which act complementary regarding co-factors, inhibitors and pH optimum [36]. We observed significantly higher DNase activity in STEMI patients than in healthy controls [24], presumably acting as a counter regulatory mechanism against DNA accumulation in acute MI. Higher culprit site DNase activity was associated with smaller infarct size in STEMI patients [30].

A SNP in the DNase 1 gene resulting in the amino acid substitution Q222R was associated with lower DNase enzymatic activity after transfection and expression in vitro [25]. The DNase 1 Q222R SNP has been linked to an increased prevalence of MI in a Japanese patient cohort [25]. The homozygous frequency of the Q222R SNP differs considerably between ethnic groups, with approximately 17% in East Asians and, 67% in Africans, and 8.7% of Europeans according to the Ensemble database [53]. We did not detect a different DNase 1 Q222R SNP frequency in our STEMI cohort (9.3%) vs. controls (8.9%).

We measured DNase activity in STEMI patients and controls, and found it lower in homozygous Q222R SNP carriers, corroborating previous observations [51]. However, despite significantly reduced DNase activity in peripheral plasma of homozygous Q222R SNP carriers suffering from STEMI, DNase activity at the culprit site was not different between genotypes.

While dsDNA and citH3 were markedly increased at the culprit site during STEMI, culprit site DNase activity was relatively low. To better describe the equilibrium of extracellular DNA and its degradation by DNase, we calculated a dsDNA/DNase ratio. The ratio was strongly increased at the culprit site, indicating excessive NET burden at the site of coronary obstruction. Furthermore, dsDNA/DNase activity ratio was significantly higher both at the culprit and the peripheral site in homozygous Q222R SNP carriers. Although the homozygous SNP is associated with decreased DNase activity, the milieu at the culprit site may play an additional important role as a modifier of DNase activity. Increased chromatin burden may be explained by substrate overload or impaired DNase enzymatic activity, presumably due to local inhibitors. Many inhibitors of DNase enzymatic activity have been described. Actin, an important contractile cardiac protein, was identified as potent endogenous DNase inhibitor, and complex formation with actin is irreversible [3]. In acute myocardial infarction, actin is released into the circulation due to inflammatory and cardiomyocyte cell death [2]. Especially at the culprit site, increased levels of actin are expected to block DNase activity by irreversible complex formation. Consequently, the degree of genetically reduced capacity of degradation could be masked by the milieu of the culprit site, potentially explaining why we could not observe a difference at the culprit site between patients homozygous for Q222R and those not. Whether release of actin or other compounds at the culprit site interferes with DNase activity remains to be determined.

We determined the impact of the Q222R SNP on outcome after STEMI in multivariable regression models. Over a mean follow-up period of 60 months, cardiovascular as well as all-cause mortality were significantly increased in homozygous SNP carriers. In concordance, dsDNA/DNase activity ratio independently predicted cardiovascular and all-cause mortality after STEMI. Our results suggest that extracellular chromatin and its degradation products are predictors of survival in patients after STEMI, while dsDNA, citH3, NE, MPO, DNase and dsDNA/DNase ratio did not correlate with enzymatic infarct size and STR. We can only speculate on the reasons behind this discordance, and we also can only speculate on the predictive power of dsDNA and the dsDNA / DNase ratio, because our data do not allow detailed analyses of causes of death. Regarding cardiovascular mortality, progression of coronary artery disease or ischemic cardiomyopathy may contribute to death. Another reason for the disconnect may be the presence of shared risk factors between cardiovascular disease and malignancy. We know that a chronic inflammatory state is deleterious to human health. Non-degraded extracellular DNA appears to be one noxious effector in an inflammatory milieu. DNases are its natural antagonists. It will be interesting to investigate the role of circulating DNA and DNases in human disease.

Differences in DNase activity appear relatively small. However, our DNase activity assay could not distinguish between different DNases; therefore, we cannot exclude that other DNases may compensate for a deficiency of DNase 1. In STEMI, circulating DNase activity is elevated in all patients, while at the culprit site, inhibitors may play a role. Apart from cardiovascular and all-cause death, no information was available on other important outcome measures, including recurrent myocardial infarction, heart failure, stroke or malignancy.

We believe that current evidence on extracellular chromatin and its efficient removal highlight it as a novel therapeutic target in cardiovascular disease. Targeting DNA in rodent MI models reduced infarct size [47]. Our present data suggest that lowering extracellular chromatin might be a promising therapeutic concept in acute Ml. Recent large-scale studies employing colchicine, an inhibitor of NET formation [41] lowering event rates after MI [44] and in chronic coronary artery disease [37] lend support to this idea. DNase 1 enzyme replacement therapy in selected patients might be a direct method to neutralize circulating DNA.

## Supplementary Information

Below is the link to the electronic supplementary material.

Supplementary file1 (DOCX 378 kb)

Supplementary file2 (JPG 43 kb)

Supplementary file3 (JPG 146 kb)

Supplementary file4 (JPG 40 kb)

Supplementary file5 (JPG 116 kb)

## Data Availability

Raw data and detailed protocols are available from the corresponding author upon request.
