# Supplementary figures and images for: Deoxyribonuclease 1 Q222R single nucleotide polymorphism and long-term mortality after acute myocardial infarction
Source: Basic Res Cardiol. 2021 Apr 23;116(1):29. doi: 10.1007/s00395-021-00864-w (PMC8064981; doi:10.1007/s00395-021-00864-w)

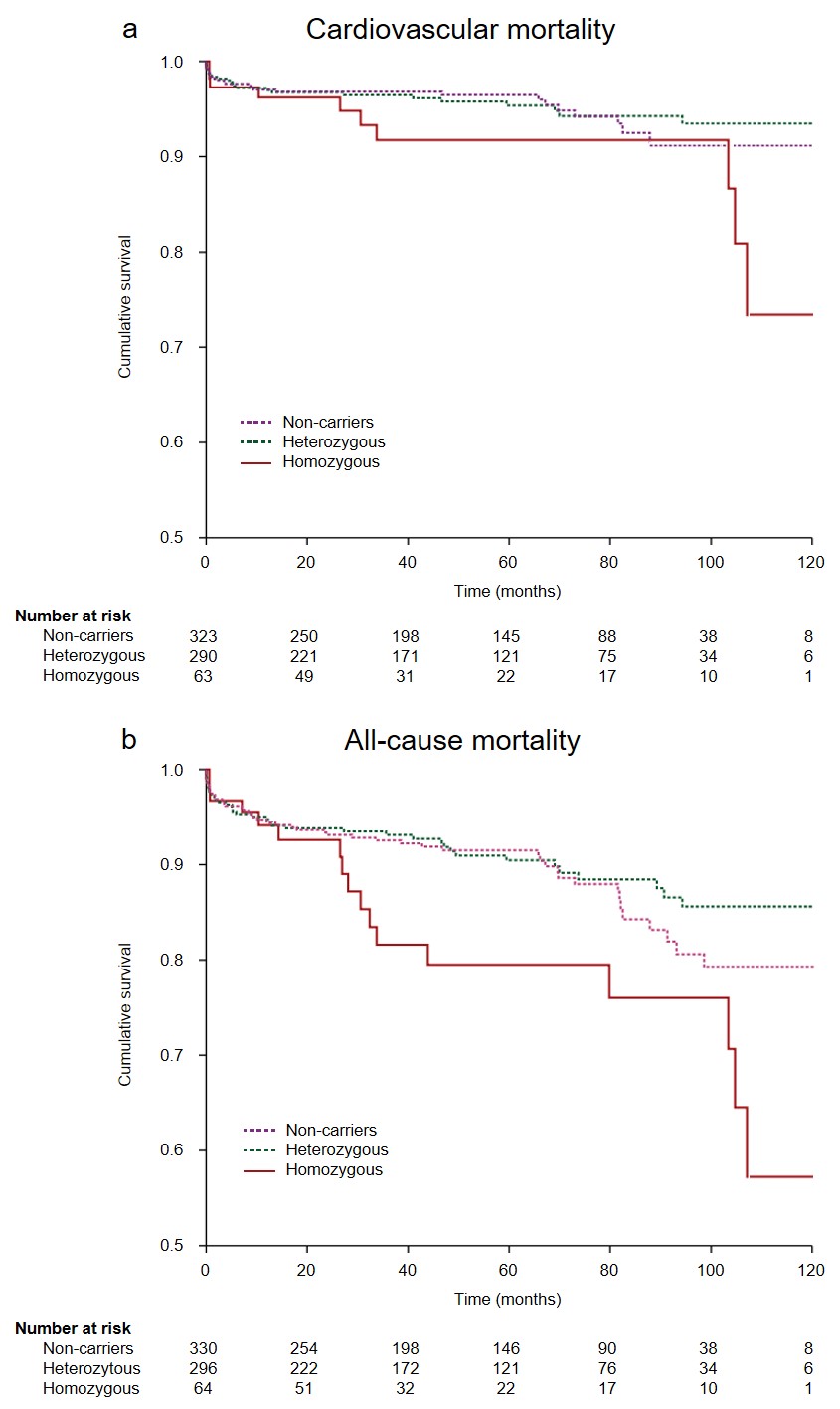

Supplement: Supplementary file 1 — Supplementary file1 (DOCX 378 kb) [file 395_2021_864_MOESM1_ESM.jpg]

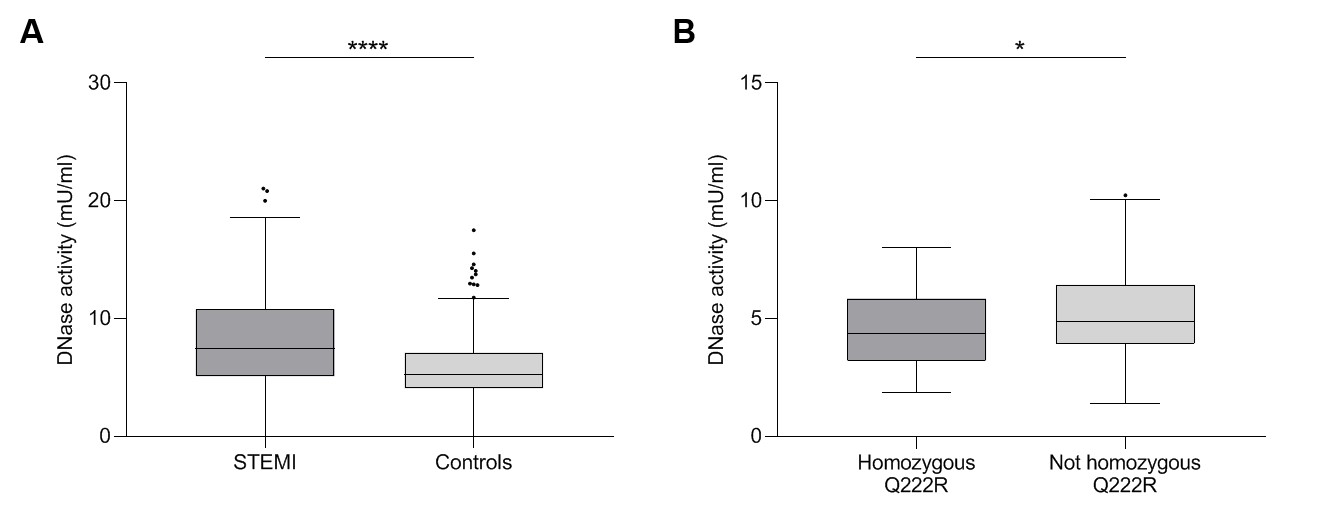

Supplement: Supplementary file 2 — Supplementary file2 (JPG 43 kb) [file 395_2021_864_MOESM2_ESM.jpg]

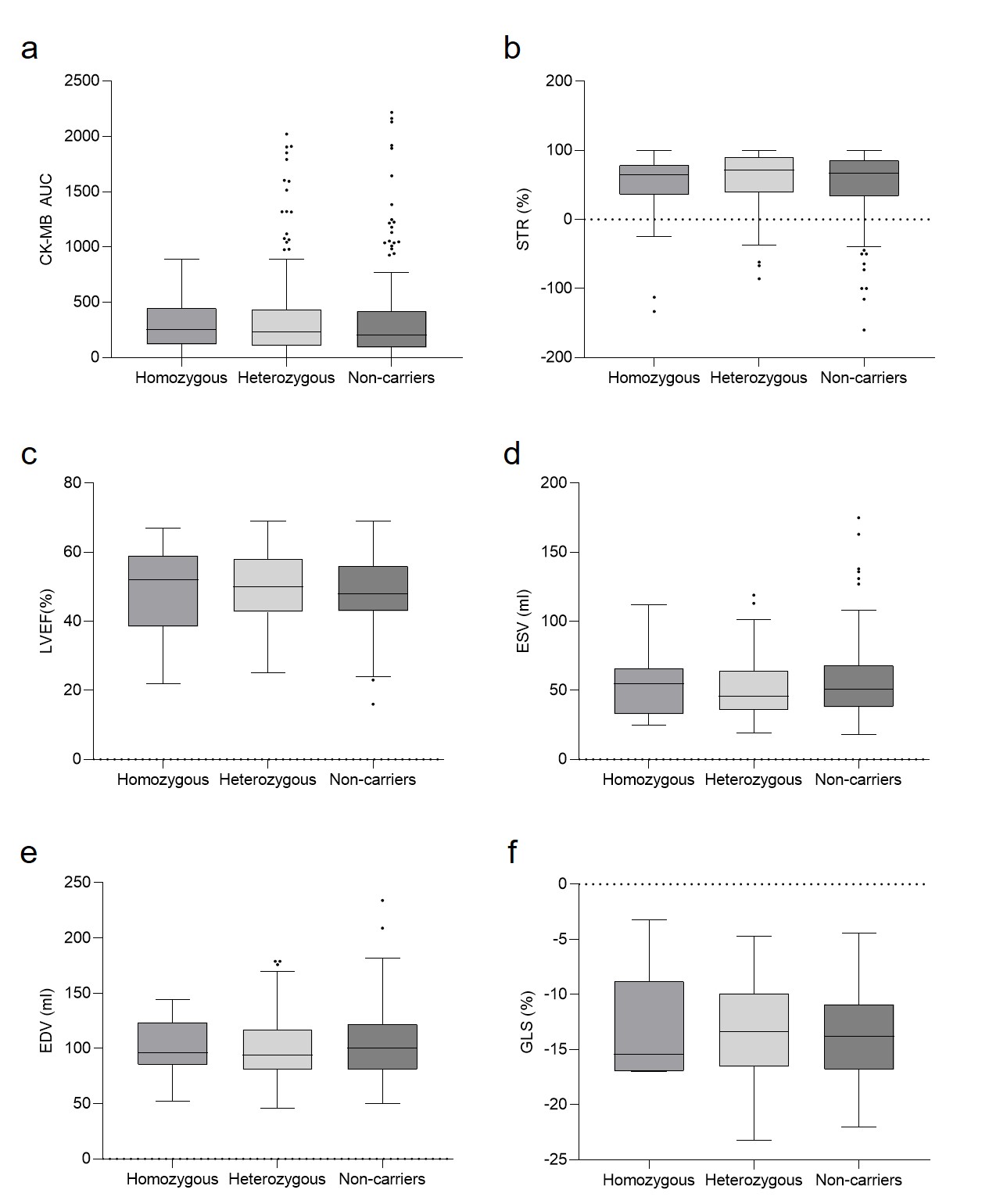

Supplement: Supplementary file 3 — Supplementary file3 (JPG 146 kb) [file 395_2021_864_MOESM3_ESM.jpg]

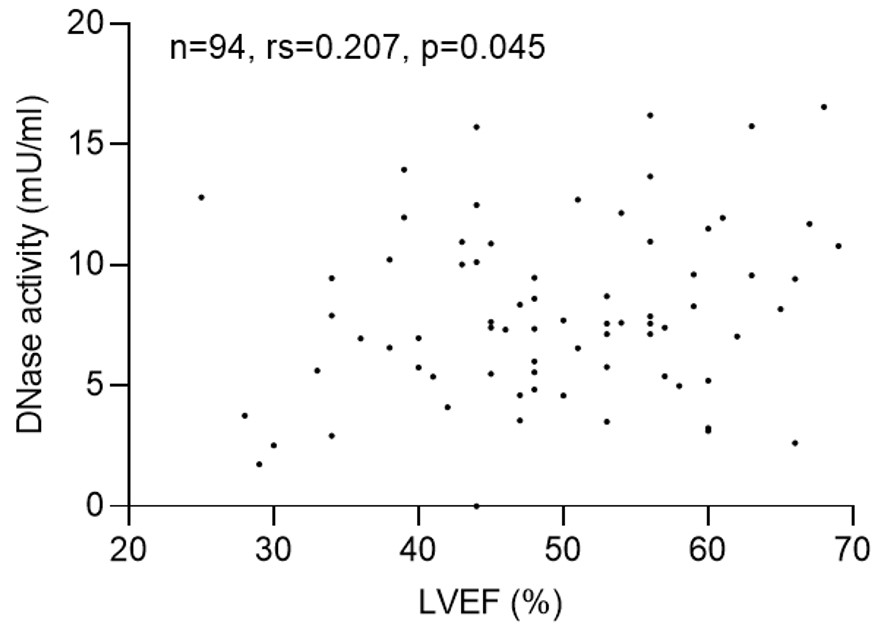

Supplement: Supplementary file 4 — Supplementary file4 (JPG 40 kb) [file 395_2021_864_MOESM4_ESM.jpg]
