# Supplementary material for: Deoxyribonuclease 1 Q222R single nucleotide polymorphism and long-term mortality after acute myocardial infarction
Source: Basic Res Cardiol. 2021 Apr 23;116(1):29. doi: 10.1007/s00395-021-00864-w (PMC8064981; doi:10.1007/s00395-021-00864-w)
Supplement: Supplementary file 5 — Supplementary file5 (JPG 116 kb) [file 395_2021_864_MOESM5_ESM.docx]

# **Supplementary Material**

**Title:** Deoxyribonuclease 1 Q222R single nucleotide polymorphism and long-term mortality after acute myocardial infarction

**Authors:** Thomas M Hofbauer ^1^†; Andreas Mangold ^1^†; Anna S Ondracek ^1^; Adelheid Panzenböck ^1^; Thomas Scherz ^1^; Julian Müller ^1^; Klaus Distelmaier ^1^; Veronika Seidl ^1^, Stefan Kastl ^1^, Martina Müller‑Nurasyid ^2,3,4^, Annette Peters ^5^; Konstantin Strauch ^2,6,7^; Robert Winker ^8^; Evelyne Wohlschläger‑Krenn ^8^; Sonja Nistler ^8^, Irene M Lang ^1^*

**Submission to**: Basic Research in Cardiology

**Corresponding author:** Irene Lang, MD, Professor of Vascular Biology, Department of Internal Medicine II, Division of Cardiology, Medical University of Vienna, Währinger Gürtel 18-20, 1090 Vienna, Austria, phone: +43-1-40400-46140, fax: +43-1-40400-42160, email: [irene.lang@meduniwien.ac.at](mailto:irene.lang@meduniwien.ac.at)

## Supplementary Table 1 Baseline characteristics of healthy controls for experimental analyses

| Patient characteristics (n=239) |  |  |
| --- | --- | --- |
| Age­­ in years, median [IQR] | | 53 [43, 61] |
| Male sex, % | | 46.9 |
| BMI, median [IQR] | | 25.2 [22.3, 29.2] |
| BMI > 25 kg/m^2^, % | | 51.3 |
| BMI > 30 kg/m^2^, % | | 20.6 |
| Diabetes, % | | 2.5 |
| History of hypertension, % | | 31.8 |
| Dyslipidemia, % | | 44.8 |
| Ever smoker, % | | 41.4 |
| Family history of CAD, % | | 20.5 |
| Previous MI, % | | 0.0 |
| CRP, mg/dL (<0.5), median [IQR] | | 0.38 [0.18, 0.77] |
| Creatinine on admission, md/dL (0.7-1.3), median [IQR] | | 1.02 [0.90, 1.16] |
| Cholesterol, mg/dL (<200), median [IQR] | | 201.54 [176.45, 228.57] |
| LDL, mg/dL (<130), median [IQR] | | 120.85 [96.91, 140.93] |
| HDL, mg/dL (>55), median [IQR] | | 64.09 [52.90, 74.90] |
| Triglycerides, mg/dL (<150), median [IQR] | | 85.84 [62.83, 119.47] |

Data are given as median [IQR] or percent of patients. Values in parentheses indicate reference values. Conversion factor for SI units for CRP 10; creatinine 76.25; cholesterol, LDL and HDL 0.0259; triglycerides 0.0113. BMI body mass index, CAD coronary artery disease, HDL high-density lipoprotein, CRP C-reactive protein, IQR interquartile range, LDL low-density lipoprotein, MI myocardial infarction

## Supplementary Table 2 Correlations of NET surrogate markers at the culprit site during STEMI

| Culprit site | dsDNA | citH3 | NE | MPO |
| --- | --- | --- | --- | --- |
| dsDNA |  | n=326  r_s_=0.485  p<0.0001 | n=224  r_s_=0.500  p<0.0001 | n=224  r_s_=0.205  p=0.002 |
| citH3 | n=326  r_s_=0.485  p<0.0001 |  | n=224  r_s_=0.473  p<0.0001 | n=224  r_s_=0.004  p=0.954 |
| NE | n=224  r_s_=0.500  p<0.0001 | n=224  r_s_=0.473  p<0.0001 |  | n=226  r_s_=0.174  p=0.009 |
| MPO | n=224  r_s_=0.205  p=0.002 | n=224  r_s_=0.004  p=0.954 | n=226  r_s_=0.174  p=0.009 |  |

citH3 citrullinated histone H3, dsDNA double-stranded DNA, NE neutrophil elastase, MPO myeloperoxidase. Correlations were calculated using Spearman’s coefficient (r_s_). Alpha level 0.05.

## Supplementary Table 3 Correlations of NET surrogate markers at the peripheral site during STEMI

| Peripheral site | dsDNA | citH3 | NE | MPO |
| --- | --- | --- | --- | --- |
| dsDNA |  | n=352  r_s_=0.432  p<0.0001 | n=256  r_s_=0.449  p<0.0001 | n=256  r_s_=-0.074  p=0.238 |
| citH3 | n=352  r_s_=0.432  p<0.0001 |  | n=258  r_s_=0.295  p<0.0001 | n=258  r_s_=-0.020  p=0.754 |
| NE | n=256  r_s_=0.449  p<0.0001 | n=258  r_s_=0.295  p<0.0001 |  | n=262  r_s_=-0.017  p=0.781 |
| MPO | n=256  r_s_=-0.074  p=0.238 | n=258  r_s_=-0.020  p=0.754 | n=262  r_s_=-0.017  p=0.781 |  |

citH3 citrullinated histone H3, dsDNA double-stranded DNA, NE neutrophil elastase, MPO myeloperoxidase. Correlations were calculated using Spearman’s coefficient (r_s_). Alpha level 0.05.

## Supplementary Table 4 NET surrogate markers, indicators of infarct size, and cardiac function after STEMI, subdivided into two Q222R DNase 1 SNP genotypes

| Parameter | Homozygous | Not homozygous | p-value |
| --- | --- | --- | --- |
| Peripheral site |  |  |  |
| dsDNA, ng/ml, median [IQR] | 164.4 [124.7, 223.3] | 153.5 [123.2, 235.4] | 0.732 |
| citH3, ng/ml, median [IQR] | 461.1 [143.6, 1394.2] | 358.3 [120.5, 1320.0] | 0.337 |
| NE, ng/ml, median [IQR] | 27.2 [11.4, 87.4] | 43.0 [21.4, 85.1] | 0.291 |
| MPO, ng/ml, median [IQR] | 221.9 [196.6, 238.8] | 234.5 [208.4, 251.5] | 0.050 |
| DNase activity, mU/ml, median [IQR] | 6.8 [4.4, 8.7] | 7.9 [5.3, 11.2] | 0.047 |
| dsDNA/DNase (ng/mU), median [IQR] | 26.3 [20.3, 54.3] | 22.5 [12.7, 42.2] | 0.031 |
| Culprit site |  |  |  |
| dsDNA, ng/ml, median [IQR] | 280.6 [199.5, 388.8] | 262.1 [169.6, 497.9] | 0.647 |
| citH3, ng/ml, median [IQR] | 1430.2 [478.4, 3124.2] | 884.8 [229.4, 2336.9] | 0.051 |
| NE, ng/ml, median [IQR] | 120.0 [64.6, 273.1] | 114.3 [60.4, 219.1] | 0.447 |
| MPO, ng/ml, median [IQR] | 244.5 [198.5, 282.8] | 266.0 [228.7, 313.9] | 0.042 |
| DNase activity, mU/ml, median [IQR] | 5.5 [3.9, 9.7] | 7.6 [5.0, 10.6] | 0.099 |
| dsDNA/DNase (ng/mU), median [IQR] | 50.7 [31.7, 84.8] | 39.4 [20.5, 73.5] | 0.027 |
| Cardiac function parameters |  |  |  |
| CK-MB AUC, median [IQR] | 251.6 [117.3, 445.7] | 170.6 [86.1, 318.0] | 0.307 |
| STR, %, median [IQR] | 64.3 [35.8, 78.6] | 72.7 [47.5, 94.4] | 0.428 |
| LVEF, %, median [IQR] | 52 [39, 59] | 50 [45, 55] | 0.906 |
| ESV, ml, median [IQR] | 55 [33, 66] | 49 [37, 63] | 0.858 |
| EDV, ml, median [IQR] | 96 [85, 124] | 100 [81, 117] | 0.877 |
| GLS, %, median [IQR] | -15.4 [-17.0, -8.8] | -14.9 [-17.0, -11.8] | 0.980 |

Data are given as median [IQR]. AUC area under the curve, citH3 citrullinated histone H3, CK-MB creatine-phosphokinase isoform MB, DNase deoxyribonuclease, dsDNA double-stranded DNA, EDV end-diastolic volume, ESV end-systolic volume, GLS global longitudinal strain, LVEF left ventricular ejection fraction, STR ST-segment resolution. Two-sided Mann Whitney test, alpha-level 0.05.

## Supplementary Table 5 NET surrogate markers, indicators of infarct size, and cardiac function after STEMI, subdivided into the three Q222R DNase 1 SNP genotypes

| Parameter | Homozygous | Heterozygous | Non-carriers | p-value |
| --- | --- | --- | --- | --- |
| Peripheral site |  |  |  |  |
| dsDNA, ng/ml, median [IQR] | 164.4 [124.7, 223.3] | 157.1 [125.8, 260.7] | 147.8 [120.7, 220.7] | 0.438 |
| citH3, ng/ml, median [IQR] | 461.1 [143.6, 1394.2] | 421.5 [141.5, 1605.5] | 283.9 [94.7, 1222.0] | 0.216 |
| NE, ng/ml, median [IQR] | 27.2 [11.4, 87.4] | 43.6 [23.5, 90.6] | 41.6 [17.2, 80.2] | 0.464 |
| MPO, ng/ml, median [IQR] | 221.9 [196.6, 238.8] | 236.9 [211.0, 255.9] | 232.8 [207.1, 248.9] | 0.057 |
| DNase activity, mU/ml, median [IQR] | 6.8 [4.4, 8.7] | 8.8 [5.9, 11.4] | 7.3 [5.1, 11.0] | 0.046 |
| dsDNA/DNase (ng/mU), median [IQR] | 26.3 [20.3, 54.3] | 21.4 [12.8, 41.8] | 23.3 [12.5, 43.0] | 0.095 |
| Culprit site |  |  |  |  |
| dsDNA, ng/ml, median [IQR] | 280.6 [199.5, 388.8] | 259.5 [158.8, 511.8] | 262.1 [172.3, 485.0] | 0.838 |
| citH3, ng/ml, median [IQR] | 1430.0 [478.4, 3123.0] | 1149.0 [256.7, 2555.0] | 717.7 [213.3, 2252.0] | 0.108 |
| NE, ng/ml, median [IQR] | 120.0 [64.6, 273.1] | 123.2 [66.4, 200.0] | 96.6 [55.8, 246.0] | 0.637 |
| MPO, ng/ml, median [IQR] | 244.5 [198.5, 282.8] | 265.2 [221.8, 307.4] | 270.0 [233.6, 318.1] | 0.110 |
| DNase activity, mU/ml, median [IQR] | 5.5 [3.9, 9.7] | 8.2 [5.0, 10.5] | 7.1 [5.0, 10.9] | 0.167 |
| dsDNA/DNase (ng/mU), median [IQR] | 50.7 [31.7, 84.8] | 31.4 [18.9, 71.3] | 43.1 [22.2, 75.1] | 0.028 |
| Cardiac function parameters |  |  |  |  |
| CK-MB AUC, median [IQR] | 251.6 [117.3, 445.7] | 233.9 [102.7, 435.1] | 202.3 [90.5, 417.8] | 0.314 |
| STR, %, median [IQR] | 64.3 [35.8, 78.6] | 71.3 [38.8, 90.1] | 66.7 [33.9, 85.7] | 0.347 |
| LVEF, %, median [IQR] | 52 [39, 59] | 50 [43, 58] | 48 [43, 56] | 0.576 |
| ESV, ml, median [IQR] | 55 [33, 66] | 46 [36, 64] | 51 [38, 68] | 0.337 |
| EDV, ml, median [IQR] | 96 [85, 124] | 94 [81, 117] | 100 [81, 122] | 0.491 |
| GLS, %, median [IQR] | -15.4 [-17.0, -8.8] | -13.4 [-16.6, -9.9] | -13.8 [-16.8, -10.9] | 0.879 |

Data are given as median [IQR]. AUC area under the curve, citH3 citrullinated histone H3, CK-MB creatine-phosphokinase isoform MB, DNase deoxyribonuclease, dsDNA double-stranded DNA, EDV end-diastolic volume, ESV end-systolic volume, GLS global longitudinal strain, LVEF left ventricular ejection fraction, STR ST-segment resolution. One-way analysis of variance with Dunn’s multiple post-hoc comparison, alpha-level 0.05.

## Supplementary Fig. 1 Measurement of DNase activity in controls without history of MI


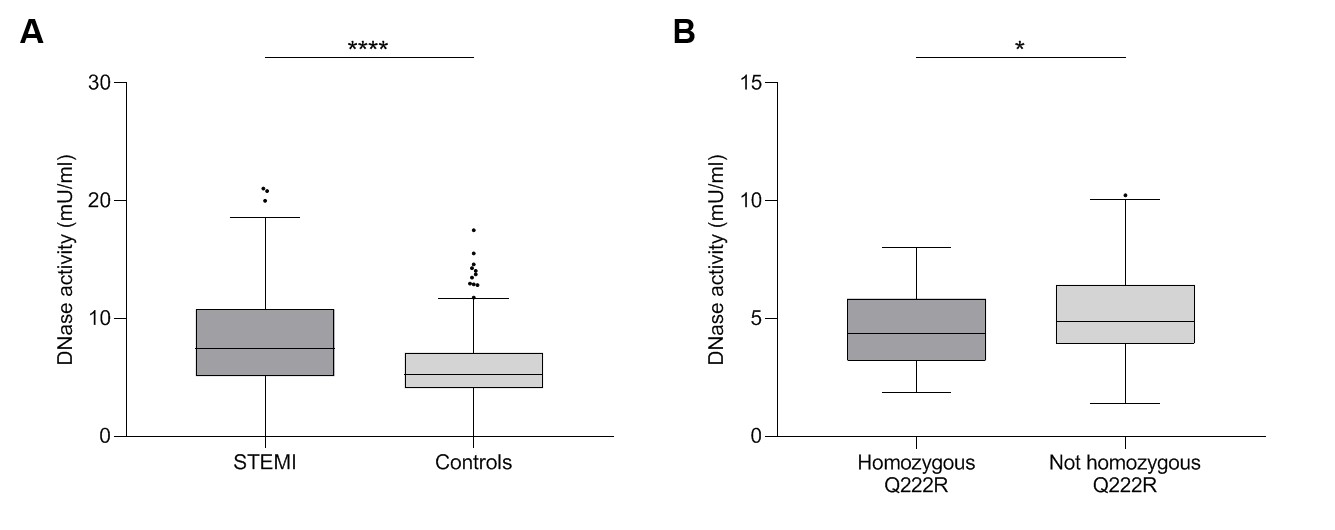


DNase activity, as measured using single radial enzyme diffusion technique, in **a**, comparison of DNase activity between STEMI patients and controls (STEMI n=262, 8.09 [5.28, 11.49] vs. controls n=239, 4.38 [3.82, 6.32] mU/ml, p<0.0001). **b**, controls without history of MI, depending on the presence of a homozygous Q222R SNP (homozygous Q222R n=34, 4.37 [3.21, 5.86] vs. not homozygous Q222R n=205, 4.89 [3.93, 6.43] mU/ml, p=0.0265). DNase deoxyribonuclease, SNP single nucleotide polymorphism, STEMI ST-segment elevation myocardial infarction. * p<0.05, **** p<0.0001. Two-sided Mann Whitney test, alpha-level 0.05.

## Supplementary Fig. 2 Comparison of markers of infarct size, and cardiac function after STEMI between the three Q222R DNase 1 SNP genotypes


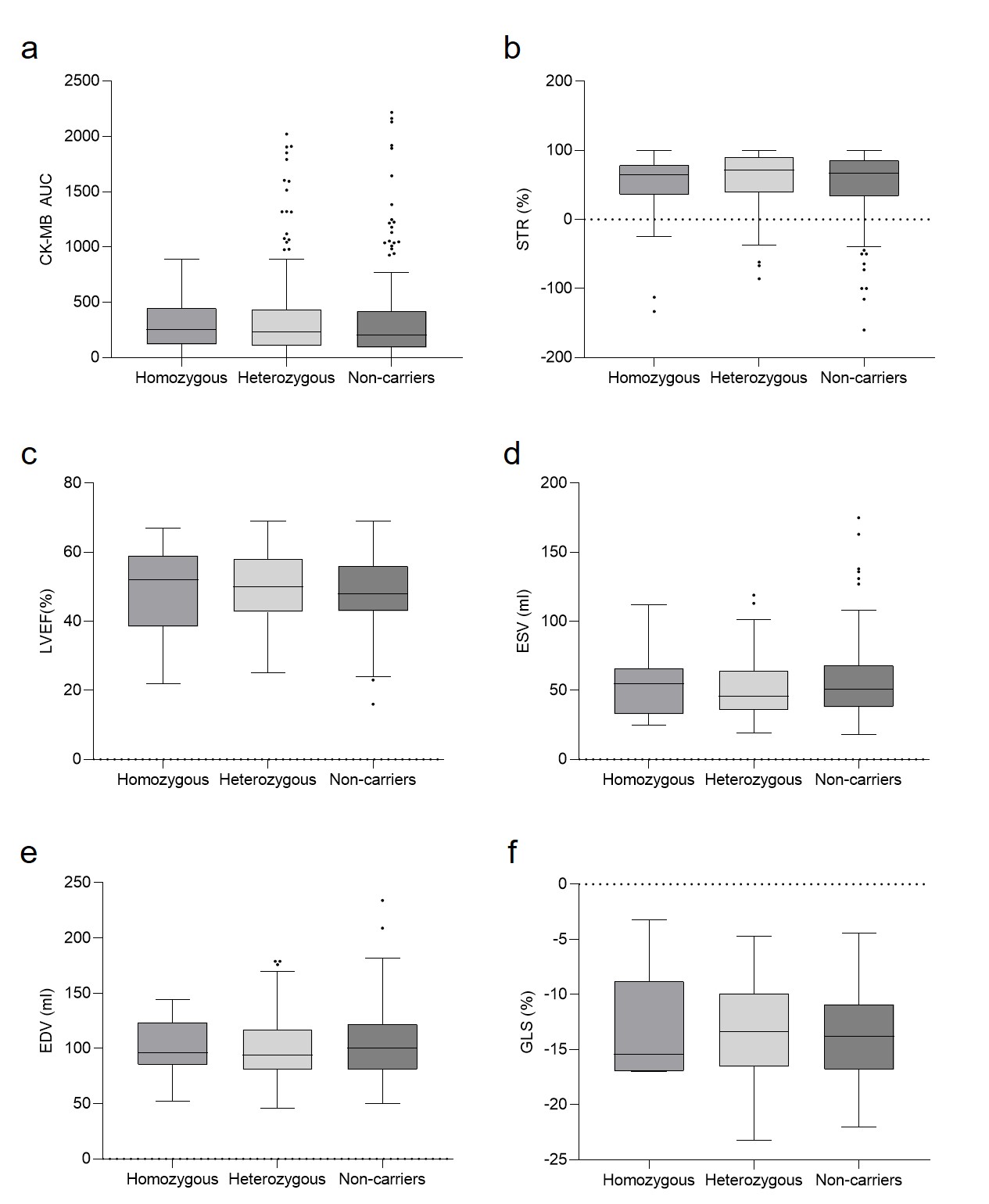


**a**, CK-MB AUC. **b**, STR. **c**, LVEF. **d**, ESV. **e**, EDV. **f** GLS. Detailed numerical analyses are provided in Supplementary Table 3. AUC area under the curve, CK-MB creatine-phosphokinase isoform MB, EDV end-diastolic volume, ESV end-systolic volume, LVEF left ventricular ejection fraction, GLS global longitudinal strain, STR ST-segment resolution. One-way analysis of variance with Dunn’s multiple post-hoc comparison, alpha-level 0.05.

## Supplementary Fig. 3 Correlation of culprit site DNase activity with LVEF


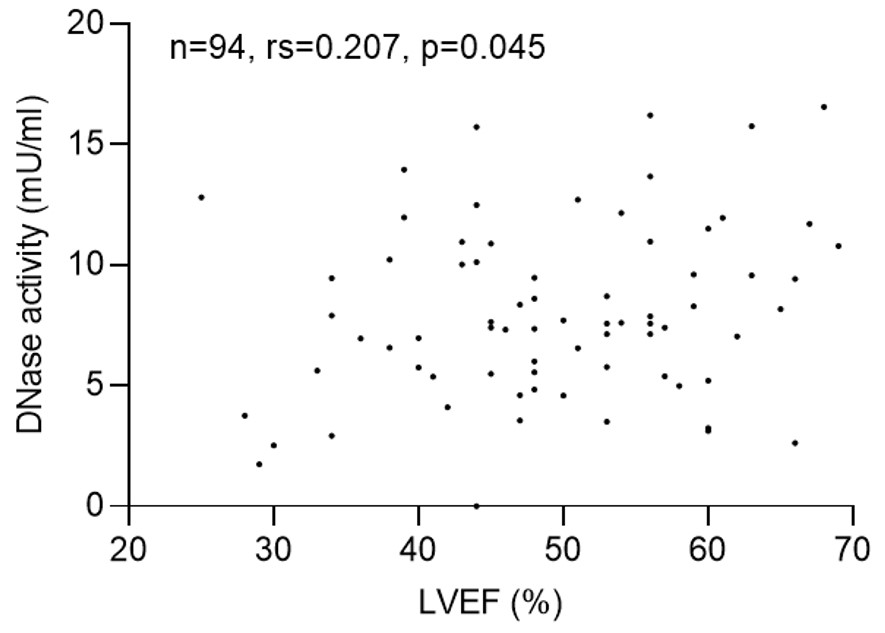


DNase activity, as measured using single radial enzyme diffusion technique, was correlated with LVEF measured using transthoracic echocardiography 3 [2, 4] days after STEMI. DNase deoxyribonuclease, LVEF left ventricular ejection fraction, STEMI ST-segment elevation myocardial infarction. Spearman’s correlation coefficient, alpha-level 0.05.

## Supplementary Fig. 4 Cardiovascular and all-cause mortality of patients after STEMI according to Q222R DNase 1 SNP carrier status


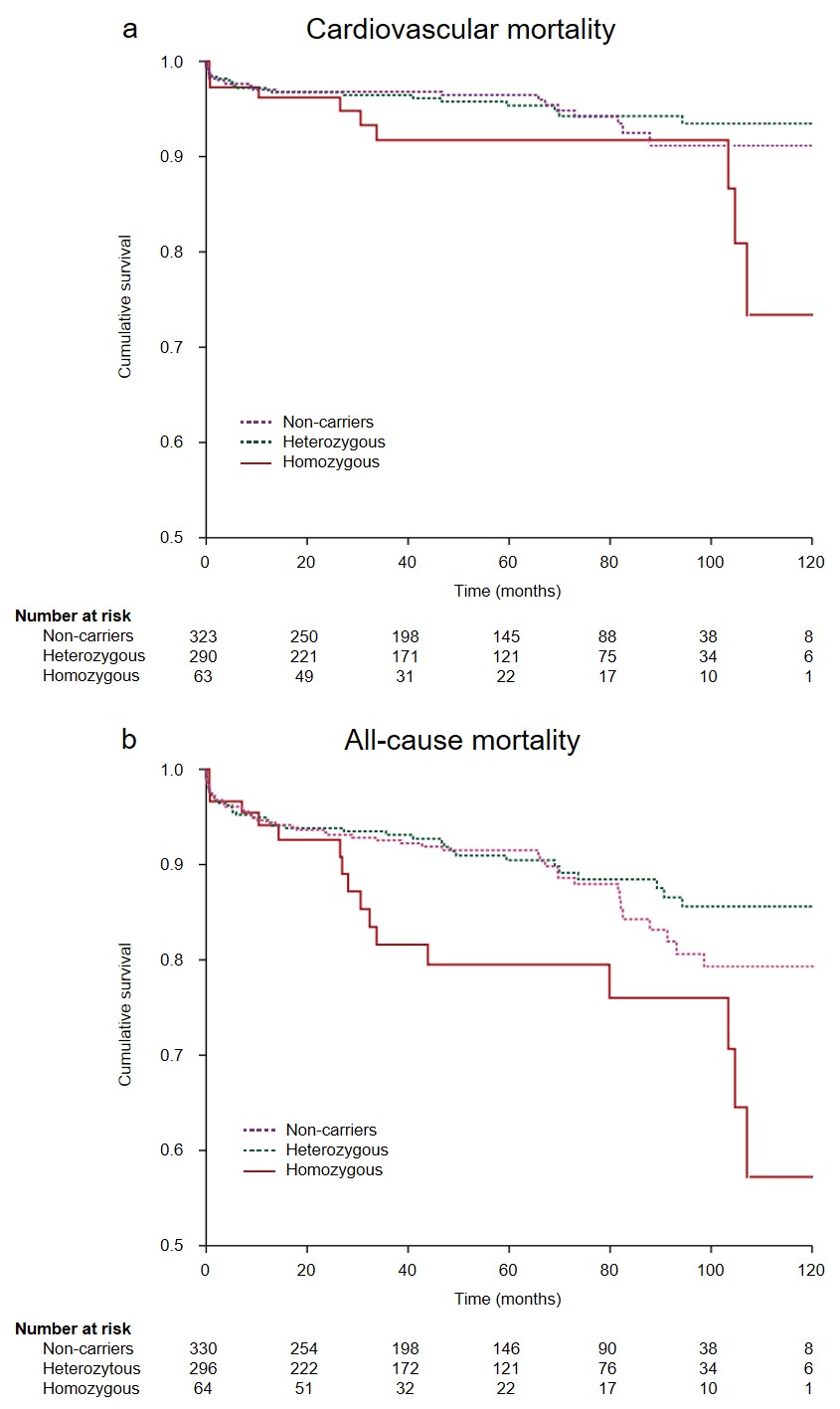


Kaplan-Meier curves depicting the influence of the homozygous Q222R DNase 1 SNP, a heterozygous variant, or non-carriers, on **a**, cardiovascular and **b**, all-cause mortality after STEMI. Censored patients are not shown. DNase deoxyribonuclease, SNP single nucleotide polymorphism, STEMI ST-segment elevation myocardial infarction.
